# Intricating connections: the role of ferroptosis in systemic lupus erythematosus

**DOI:** 10.3389/fimmu.2025.1534926

**Published:** 2025-02-04

**Authors:** Guowang Zhao, Xinghai Li, Ying Zhang, Xingzi Wang, Li Deng, Juan Xu, Shumei Jin, Zan Zuo, Linting Xun, Mei Luo, Fan Yang, Jialong Qi, Ping Fu

**Affiliations:** ^1^ Department of Rheumatology and Clinical Immunology, The Second Affiliated Hospital of Kunming Medical University, Kunming, Yunnan, China; ^2^ Department of Minimal Invasive Intervention Radiology, Ganzhou People’s Hospital, Ganzhou, Jiangxi, China; ^3^ Yunnan Digestive Endoscopy Clinical Medical Center, Department of Gastroenterology, The First People’s Hospital of Yunnan Province, Affiliated by Kunming University of Science and Technology, Kunming, Yunnan, China; ^4^ School of Medicine, The First People’s Hospital of Yunnan Province, Kunming University of Science and Technology, Kunming, Yunnan, China; ^5^ Department of Nephrology, Yueyang Central Hospital, Yueyang, Hunan, China; ^6^ Department of Internal Medicine, Community Health Service Station of Dian Mian Avenue, Kunming, Yunnan, China; ^7^ Yunnan Institute of Food and Drug Supervision and Control, Medical Products Administration of Yunnan Province, Kunming, Yunnan, China; ^8^ School of Medicine, Kunming University of Science and Technology, Kunming, Yunnan, China; ^9^ Yunnan Provincial Key Laboratory of Clinical Virology, The First People’s Hospital of Yunnan Province, Kunming, Yunnan, China; ^10^ Yunnan Provincial Key Laboratory of Birth Defects and Genetic Diseases, First People’s Hospital of Yunnan Province, Kunming, Yunnan, China

**Keywords:** systemic lupus erythematosus (SLE), ferroptosis, interferons (IFNs), mitochondrial, damage-associated molecular patterns (DAMPs)

## Abstract

Systemic lupus erythematosus (SLE) is a chronic inflammatory and autoimmune disease with multiple tissue damage. However, the pathology remains elusive, and effective treatments are lacking. Multiple types of programmed cell death (PCD) implicated in SLE progression have recently been identified. Although ferroptosis, an iron-dependent form of cell death, has numerous pathophysiological features similar to those of SLE, such as intracellular iron accumulation, mitochondrial dysfunction, lipid metabolism disorders and concentration of damage associated-molecular patterns (DAMPs), only a few reports have demonstrated that ferroptosis is involved in SLE progression and that the role of ferroptosis in SLE pathogenesis continues to be neglected. Therefore, this review elucidates the potential intricate relationship between SLE and ferroptosis to provide a reliable theoretical basis for further research on ferroptosis in the pathogenesis of SLE.

## 1 Introduction

Systemic lupus erythematosus (SLE) is a chronic systemic autoimmune disease characterized by inflammation and immune-mediated injury to various organs and systems. Approximately 3.4 million people are affected by SLE, with approximately 400,000 people diagnosed with SLE each year worldwide (1, 2). Despite the diversity of clinical manifestations and the extremely complex pathogenesis in SLE, high levels of interferons (IFNs) and autoantibodies are common features (3), which also are primarily the result of cell death. In SLE, excessive cell death and impaired clearance machinery contribute to the continuous release of intracellular nucleic acids and their complexes into the extracellular space, thereby promoting responses by autoreactive B cells and IFN-responsive mechanisms (4). Interestingly, some prevalent features of SLE, including iron overload, mitochondrial dysfunction, lipid metabolism disorders and damage associated molecular pattern (DAMP) concentrations, are complexly associated with cell death, especially ferroptosis (5, 6).

Ferroptosis is an iron-dependent immunogenic form of cell death that is distinct from cell death mediated by perforin rupture (7). The occurrence of ferroptosis is attributed mainly to an imbalance in intracellular oxidation and antioxidant mechanisms, which triggers a cascade of hydrogen peroxide-lipid reactions and mediates the rupture of the cell membrane. Its mechanism mainly includes lipid peroxidation (mainly involving iron homeostasis and mitochondrial dysfunction) and antioxidant system disorders [mainly involving the cystine/glutamate transport system (xCT), glutathione (GSH)-glutathione peroxidase 4 (GPX4)] (5). Ferroptosis is induced by many factors, such as excessive DAMPs [IFN-α, IFN-γ, high-mobility group box 1 (HMGB1)] (8), ionizing radiation (9), the accumulation of iron, and increases in free radicals and oxidized lipids (10). Although the physiological role of ferroptosis is still unclear, its pathological role in a variety of acute and chronic diseases has been widely reported (11, 12), and it is potentially closely related to the pathogenesis of SLE (13, 14).

To date, the pathogenesis of SLE has not been classified. PCD has been shown outstanding role in SLE pathogenesis. Ferroptosis is a novel form of PCD, which exhibits several pathophysiological features reminiscent of those seen in SLE, including intracellular iron accumulation, mitochondrial dysfunction, lipid metabolism disorders, and regulation by DAMPs (**Table 1**). These similarities warrant further exploration of the potential interplay between ferroptosis and SLE pathogenesis. Although ferroptosis has been reported to play a role in SLE, the relationship between ferroptosis and SLE remains unclear. Therefore, this review discusses those parallel features between ferroptosis and SLE promote further research on ferroptosis in the pathogenesis of SLE.

**Table 1:** Parallel features in SLE and ferroptosis.

Parallel features	Key points	
	SLE	Ferroptosis
Iron accumulation	Iron accumulation is pervasive in SLE kidney and pathogenic immune cells (15)	Iron overload is a vital trigger for ferroptosis (12)
Mitochondrial dysfunction	impaired mitochondria are central mediators of injury in different tissues and organs in SLE (16)	Mitochondria serves as key organelles for triggering ferroptosis (17)
Lipid metabolism disorders	Dyslipidemia and amplification of lipid peroxides are common features in SLE (18)	Lipid peroxides is executor of ferroptosis and some enzymes and intermediates of cholesterol metabolism are regulators for ferroptosis (19)
HMGB1 concentration	HMGB1 serve as a biomarker for SLE and potential aggravates the disease progression (20)	HMGB1 serves as the main DAMP of ferroptosis and can trigger ferroptosis in neighboring cells (21–23)
Regulation by IFNs	SLE is regarded as “IFNs signature” disease (3, 24)	IFN-I/II are key regulators of ferroptosis (8)

## 2 Primary regulation in ferroptosis

Current evidence demonstrated intracellular oxidation primary related to some intracellular alterations, encompassing iron accumulation, mitochondrial dysfunction and lipid metabolism disorder (5). And antioxidant systems related to defend ferroptosis mainly including the xCT-GPX4, ferroptosis suppressor protein 1 (FSP1)-coenzyme Q10 (CoQ10), Guanosine triphosphate cyclohydrolase 1 (GCH1)-tetrahydrobiopterin (BH4) and dihydroorotate dehydrogenase (DHODH)-dihydroubiquione (CoQH2) systems (5, 25). Conceivably, biological processes related to the homeostasis of oxidation-reduction are peculiarly prone to regulate ferroptosis, such as lipogenesis (26, 27), autophagy (mainly including ferritinophagy, lipophagy and mitophagy) (28, 29), and the tricarboxylic acid (TCA) cycle (17). Moreover, plenty of and metabolic molecules are crucial for primary ferroptostic suppressors, such as: isopentenylation is required for the synthesis of selenoenzymes including GPX4 and ubiquinone (also known as coenzyme Q) is a major substrate of FSP1, both them is metabolites of mevalonate pathway (30). Additionally, multiple DAMPs can regulate ferroptosis, such as: IFNs can regulate solute carrier family 7a member 11 (SLC7A11, subunit of xCT) and GPX4 expression by activating JAK-STAT1 signaling (8); HMGB1 can induce ferroptosis by promoting autophagy and iron accumulation (31, 32); Mitochondrial DNA (mtDNA) performs ability to trigger ferroptosis by activating the cyclic GMP-AMP receptor stimulator of interferon genes (STING) (33).

## 3 Similar features in SLE and ferroptosis

### 3.1 Iron accumulation

Iron is one of the essential trace elements in the human body and is crucial for maintaining various biological functions, such as transporting oxygen, participating in the production of energy in the respiratory chain, and being involved in key biological reactions as a key component of various enzymes (34). However, iron accumulation can also mediate a series of pathophysiological processes, such as reactive oxygen species (ROS) production, lipid peroxide production and mitochondrial dysfunction, thereby promoting disease progression, such as SLE (35). Iron accumulation has been detected in the kidneys of both SLE patients and lupus mice (36, 37), and multiple molecules related to iron metabolism [such as ferritin, transferrin, ceruloplasmin, and neutrophil gelatinase-associated lipid carrier protein (NGAL)] have been identified as biomarkers of SLE and/or LN, which are associated with disease activity and/or renal involvement (38–43). Additionally, the excessive intake of iron exacerbates SLE progression (44–46), whereas iron chelation can alleviate the disease (37, 47).

Kidney is major damaged organ in SLE and main organ attacked by iron overload because it is site of iron filtration and reabsorption. Over transferrin bound (TBI) and non-transferrin bound iron (NTBI) can overwhelm the heavy chain ferritin (FtH) capacity, which lead to release of labile iron and render proximal tubular epithelial cells (PTEC) susceptible to iron oxidant damage, especial in distal tubular epithelial cells (DTEC) with lack iron storage (light and heavy chain ferritin) and export protein (ferroportin) (15). Moreover, this damage can be worse under some pathological condition like LN with glomerular injury resulting in an increased leakage of TBI and NTBI (48). Additionally, SLE is a typical autoimmune disease, and the overactivation of CD4^+^ T lymphocytes is a feature of SLE (28). In SLE, CD4^+^ T cells are over differentiated into pathogenic Th1 and Th17 cells, whereas Treg cells are suppressed (49, 50). Interestingly, iron is crucial for regulating the activation, proliferation and differentiation of CD4^+^ T lymphocytes (51). Some reports have demonstrated that the iron concentration in CD4^+^ T cells is significantly greater in SLE patients than in healthy controls (52, 53). CD71 (also referred to as ferritin receptor TfR1, which mediates iron endocytosis) on the surface of CD4^+^ T cells is essential for their activation and proliferation and promotes their differentiation into Th17 cells (54–56). Moreover, Th17 cells in SLE patients express high levels of CD71, which is positively correlated with disease activity (56). Similarly, follicular helper T (Tfh) cells, as a specific subset of CD4^+^ T cells, contribute to the pathogenesis of SLE by promoting the maturation of germinal center B cells and the production of antibodies (57, 58). However, the accumulation of iron in CD4^+^ T cells in SLE can promote their differentiation into Tfh cells and aggravate the progression of SLE (53). Moreover, Treg cells can restrict CD4^+^ T cell overactivation and its dysfunction is involved in SLE pathogenesis (59–61). Gao XF et al. demonstrated iron deficiency could alleviate pristane-induced lupus progression by promoting Treg cell expansion (62). And Feng P et al. verified iron overload leaded to systemic autoimmune disorders by aggravating Treg cell death (63). These findings verify that iron overload is involved in the pathogenesis of SLE, but whether it occurs through ferroptosis is not clear.

Iron overload is also conducive to ferroptosis. Too much iron intake, too little iron excretion, or impaired iron metabolism can contribute to iron overload. Physiologically, there are several ways to reduce the level of intracellular iron when the level of intracellular iron is excessive: i. iron-responsive proteins/iron-responsive elements (IRPs/IREs) hamper the transfer of iron by disrupting the stability of transferrin receptor mRNA while promoting the transcription and synthesis of ferritin to enhance the binding of free iron (64–66); ii. increased expression of hepcidin promotes the expression of iron transfer protein (ferroportin), which promotes iron outward transport (67–69); and iii. ferritin is targeted to lysosomes by nuclear receptor coactivator 4 (NCOA4)-mediated autophagosomes for degradation (often referred to as ferritinophagy) (70, 71). Iron overload is a vital trigger for ferroptosis. i.Iron-mediated Fenton reactions are necessary for ferroptosis and can facilitate the production of phospholipid hydroperoxides (PLOOH, a biomarker of ferroptosis). ii.Key enzymes that trigger lipid peroxidation [lipoxygenases (LOXs) and cytochrome P450 oxidoreductases (PORs)] require iron catalysis. iii.Iron is required for numerous redox-based metabolic processes and is a major source of intracellular ROS (11, 34). iv.Iron chelating agents not only prevent ferroptosis but also reduce the production of lipid peroxides (72–75). Feng P et al. reported that iron overload in Treg cells can promote iron-dependent cell death (63). Liu Y et al. shown iron overload in the joint cavity of rheumatoid arthritis patients and *in vitro* can promote ferroptosis in macrophages (76). Additionally, Alli, A. A. et al. demonstrated iron sequestration within the proximal tubules promoted LN progression by exacerbating ferroptosis (77). This evidence demonstrated that iron accumulation can trigger ferroptosis not only in the extracellular space but also in the intracellular space.

**Figure 1:**
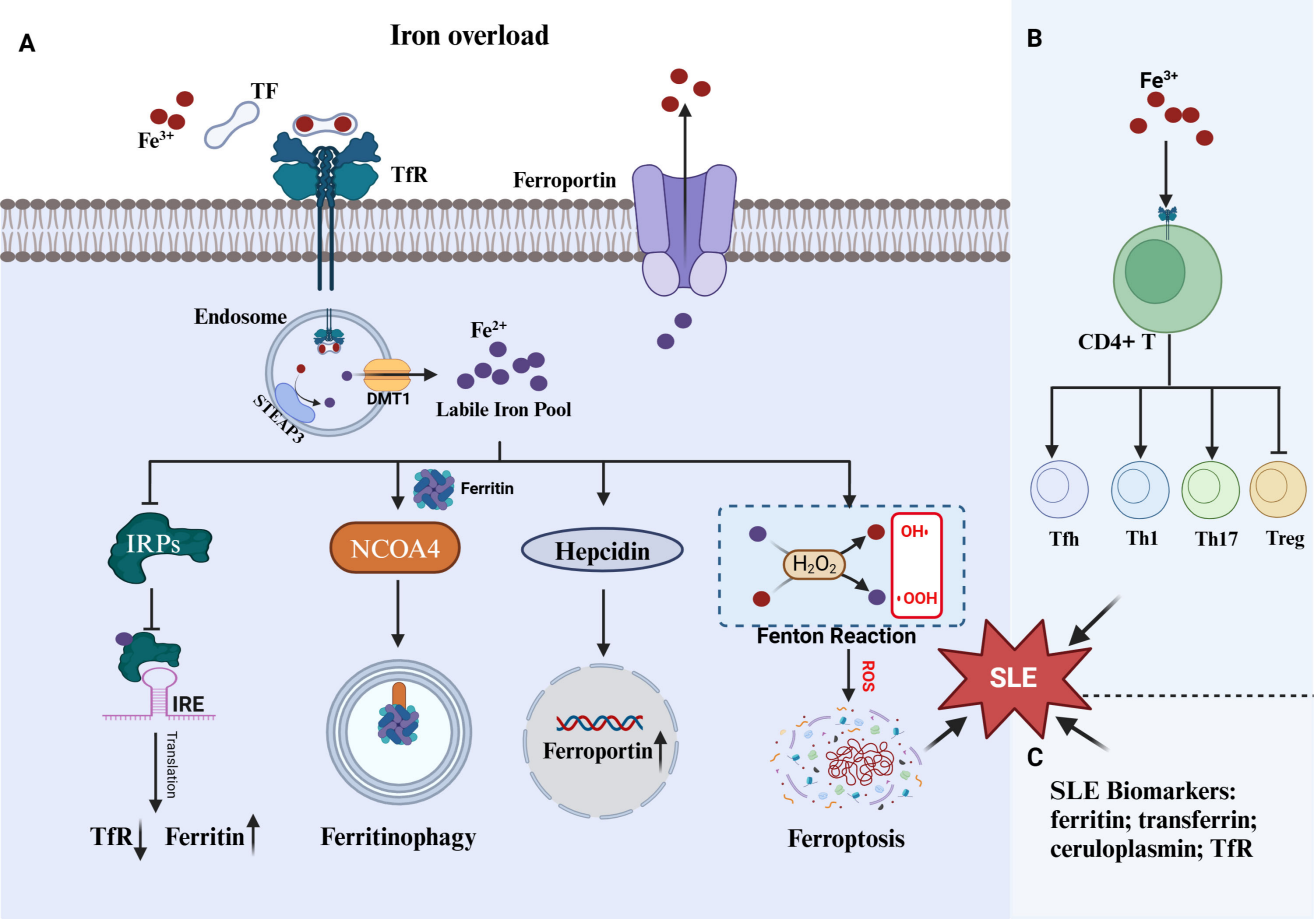
Iron overload in ferroptosis and SLE. **(A)** Three mechanism including IRPs/IRE system, ferritinophagy and hepcidin-ferroportin roadways against iron overload. The cell undergoes ferroptosis when those mechanism are impaired. **(B)** Iron overload promote CD4^+^T abnormal proliferation and differentiation, which can promote SLE progress. **(C)** Numerous serum protein closed to iron regulation were identified as SLE biomarkers. TF, transferrin; TfR, transferrin receptor; IRPs, iron-responsive proteins; IREs, iron-responsive elements; STEAP3, six-transmembrane epithelial antigen of the prostate 3; DMT1, Divalent metal transporter 1; NOCA4, nuclear receptor coactivator 4.

Thus, iron overload is a shared feature of SLE and ferroptosis and multiple evidences showed iron overload was involved in disease progression by triggering ferroptosis (**Figure 1**). Although, numerous reports demonstrated iron overload can aggravate the progression of SLE, whether the effect is ascribed to promoting ferroptosis. Anyhow, preventing iron accumulation in tissues and cells by mediating the key molecules of iron metabolism (transferrin receptor, ferroportin, hepcidin, etc.) may be a novel direction for the treatment of SLE.

### 3.2 Mitochondrial dysfunction

Mitochondria are essential organelles for maintaining the normal physiological function of the cell and play a crucial role in physiological processes such as energy production, calcium homeostasis, and iron metabolism. It is also a key site for ROS elevation, self-nucleic acid antigen production, and the promotion of various forms of cell death (such as ferroptosis), which are closely related to a variety of diseases, such as SLE (26, 78, 79). Overactivation and immune intolerance of immune cells is the most pivotal link in the pathogenesis of SLE (28). The rapid proliferation and differentiation of immune cells require mitochondria to supply a large amount of energy. Oxidative phosphorylation (OXPHOS) of the electron transport chain (ETC) on the inner mitochondrial membrane is the process of ATP production, which is also accompanied by ROS production (80). Dysfunction of mitochondria limits ATP production but aggravates the release of ROS, which are closely related to immune disorders, autoantibody production, excessive cell death and ineffective clearance mechanisms (81). Previous studies have shown that mitochondria in CD4^+^ T cells from SLE patients exhibit numerous abnormalities, such as large size (mitochondrial fusion), membrane hyperpolarization (elevated mitochondrial transmembrane potential), ATP depletion, fragility, increased mitochondrial ROS (mtROS) and decreased antioxidant (GSH) levels (82–84). Moreover, massive ROS production in SLE patients is the main contributor to mitochondrial dysfunction (85). Second, mitochondrial DNA (mtDNA) oxidized by ROS (ox-mtDNA) has been identified as an important DAMP. Under normal physiological conditions, it can be dissociated from mitochondrial transcription factor A (TFAM) and degraded in lysosomes. However, in SLE, the restriction of TFAM function promotes the continuous accumulation of Ox-mtDNA. In addition, mtDNA that is released into the cytoplasm can not only activate cyclic GMP-AMP synthase (cGAS) or Toll-like receptors 9 (TLR9) to promote the production of IFNs (86, 87) but also serve as an antigen to directly activate the immune system to exacerbate autoantibody production (81). Since the kidney is one of the most involved organs in lupus and iron consumption is prevalent in it, mitochondrial are particularly susceptible to oxidative attack in glomerular and tubular cells. Tian Y at al. Demonstrated the molecular mechanism of iron-induced injury in these cells was contributed to mtROS overproduction (88). Remarkably, recent evidence demonstrated impairment of mitochondrial degradation in glomerular and tubular cells was implicated in proteinuria and renal failure in LN (16, 89, 90). Moreover, both mtROS and impair mitochondrial degradation promote Ox-mtDNA accumulation (91). On the other side, Ox-mtDNA can exacerbate mtROS production in a vicious cycle, which further aggravate the disease progression.

Moreover, mitochondria serve as central hubs for multiple forms of cell death, such as apoptosis, necroptosis, and pyroptosis, and are also key organelles that trigger ferroptosis (5, 78, 92). It is still debated whether mitochondria are necessary for ferroptosis. For example, some cells become insensitive to ferroptosis-inducing agents after the removal of mitochondria, whereas others do not (93, 94). Moreover, it has also been shown that mitochondria are required for erastin-induced ferroptosis but not for RSL3-induced ferroptosis (17). However, deficiency of optic atrophy protein 1 (OPA1), which maintains mitochondrial homeostasis and function, leads to resistance to both erastin- and RSL3-induced ferroptosis (95). In ferroptosis, significant changes in mitochondrial function and morphology are observed, including a decrease in volume, increase in membrane density, and decrease in ridge and ATP production (7). The role of mitochondria in triggering ferroptosis is explained as follows: First, mitochondria are one of the main sources of ROS and the key site of energy production and iron metabolism (65, 96). Mitochondrial dysfunction not only reduces ATP production but also contributes ETC to leaking large amounts of ROS (such as O_2_·- and H_2_O_2_) and disturb iron homeostasis. H_2_O_2_ combines with the Fe^2+^-mediated Fenton cascade and further exacerbates the massive release of ROS, which promotes lipid peroxidation (97). Depletion of mtROS can alleviate mitochondrial ferroptosis (98). Second, the tricarboxylic acid cycle (TCA) cycle is located in the mitochondria. The mitochondrial TCA cycle can increase ferroptosis sensitivity (17). Glutamate metabolism is an important complementary part of the TCA cycle. In mitochondria, glutamine can be converted to glutamate and TCA intermediate metabolites (α-ketoglutarate, αKG), both of which can promote ferroptosis (99–101). Moreover, the inactive αKG dehydrogenase complex can prevent ferroptosis induced by cystine depletion (102). Additionally, ferroptosis has been identified as a form of autophagy-dependent cell death (103). And interesting, some reports demonstrated mtDNA could trigger autophagy-dependent ferroptosis by activating STING (33) and peroxisome proliferator activated receptor alpha (PPARα) (104). Moreover, mtDNA is prone to transform B- to Z-DNA structures under mtROS stress (105), which could promote ferroptosis by activating Z-DNA binding protein 1 (ZBP1) (106). These results suggest that mitochondrial dysfunction can promote ferroptosis.

**Figure 2:**
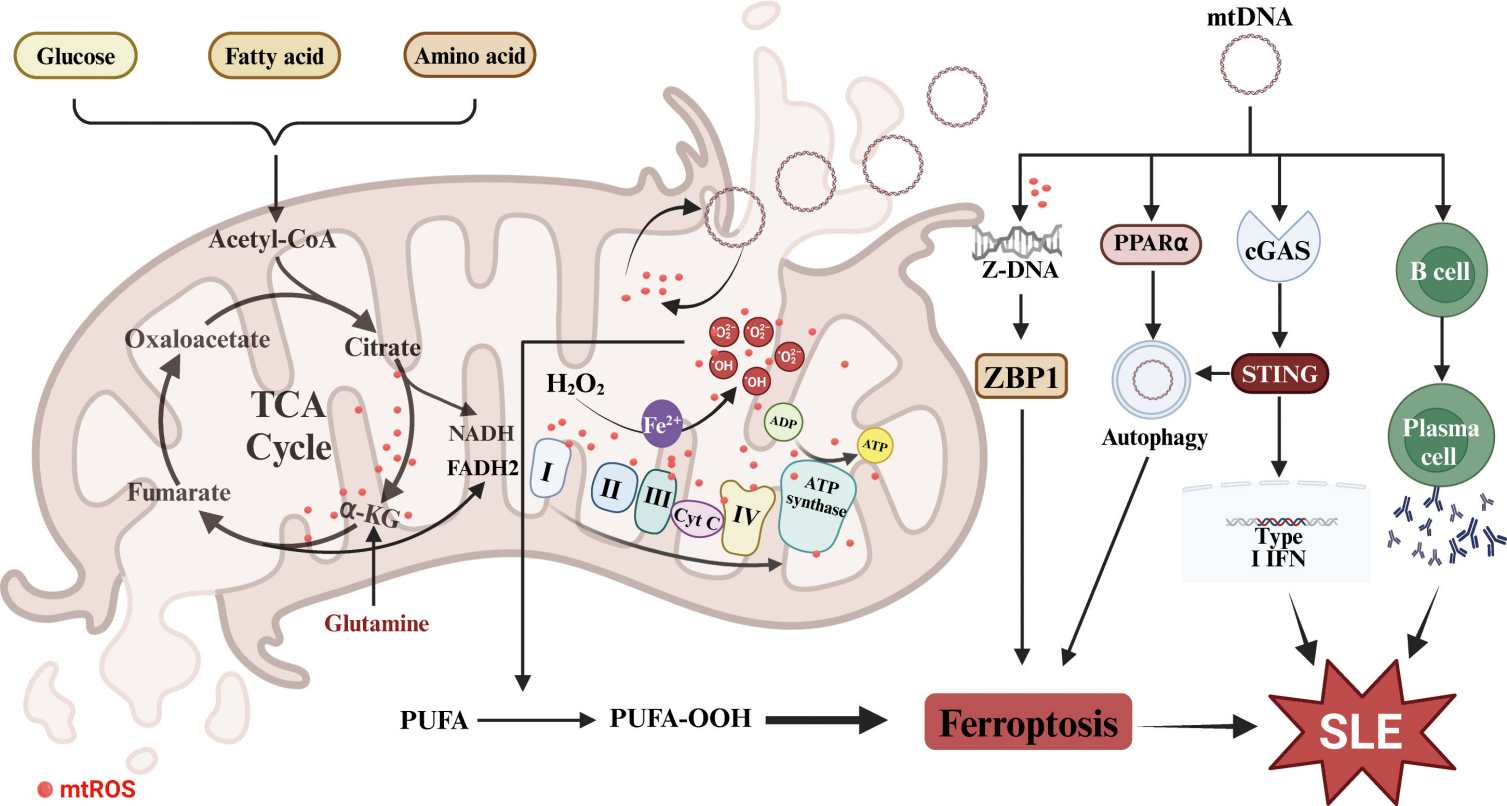
Mitochondrial dysfunction in SLE and ferroptosis. The dysfunction promotes mtROS accumulation and mtDNA release, which triggers cell ferroptosis and enhances the production of autoantibodies and IFNs. PUFA, polyunsaturated fatty acids; TCA, the tricarboxylic acid cycle; α-KG, α-ketoglutarate; mtROS, mitochondrial reactive oxygen species; mtDNA, mitochondrial DNA; ZBP1, Z-DNA binding protein 1; PPARα, peroxisome proliferator activated receptor alpha.

In summary, in SLE patients, mtROS accumulation and mtDNA release may promote ferroptosis and subsequent release of intracellular antigens and DAMPs, thereby mediating the progression of SLE (**Figure 2**). Therefore, limiting mtROS production and mtDNA release to maintain mitochondrial function may be a promising treatment strategy for SLE.

### 3.3 Lipid metabolism disorder

Lipid metabolism is a major physiological process that includes catabolism [fatty acid oxidation (FAO)], anabolism (*de novo* lipogenesis) and storage [lipid droplets (LDs)], and lipid metabolism disorders are closely related to multiple pathological processes and diseases, including immune dysfunction and SLE (107). Dyslipidemia, a major type of lipid metabolic disruption, is most common in SLE patients (68%-100%) and is characterized primarily by increased plasma levels of very low-density lipoprotein (VLDL), triglyceride (TG) and total cholesterol (TC) but decreased high-density lipoprotein (HDL) levels (108). This disorder not only is closely related to SLE activity and organ involvement (including the cardiovascular system and kidney) but is also related to high mortality (109, 110). Among them, low HDL-cholesterol (HDL-c) levels are the most common sign of dyslipidemia in SLE patients (18, 111, 112). Under normal conditions, HDL performs anti-inflammatory and antioxidative functions despite its cholesterol efflux capacity. However, in SLE, it functions in an opposite manner with an uncertain mechanism (113, 114). Interestingly, a previous study demonstrated that limiting cholesterol flux could aggravate the IFN and ISG response in a STING-dependent manner in macrophages (115), and its precursor concentration, 7-dehydrocholesterol (7-DHC), could also significantly increase I-IFN production via the phosphatidylinositol 3-kinase (PI3K)-protein kinase B 3 (AKT3)-interferon regulatory factor 3 (IRF3) pathway (116). However, 7-DHC, as a precursor to form Vitamin D3 in the skin by solar ultraviolet B radiation, is the main source of Vitamin D. The deficiency Vitamin D is prevailed in SLE population and its supplementation seems to ameliorated the diseases (117), which may attribute that adequate level of Vitamin D prevents the synthesis of 7-DHC. Additionally, the metabolite of cholesterol, 7α, 25-dihydroxycholesterol (7α, 25-OHC), is dramatically elevated in the plasma of SLE patients and binds to the G protein-coupled receptor (GPCR) Epstein–Barr virus-induced gene 2 (EBI2) in macrophages to alleviate the IFN and ISG response for protecting against SLE progress (118). Consequently, homeostasis lipidemia, especially cholesterol metabolism, is crucial for SLE pathogenesis.

Additionally, lipids are highly vulnerable to oxidation, and many lipid peroxides, including oxidized HDL (ox-HDL), ox-LDL, malondialdehyde (MDA) and 4-hydroxynonenal (4-HNE), have been identified as potential biomarkers for SLE and are related to disease activity (111, 118–121). Because oxidative stress is involved in the pathogenesis of SLE, aberrant oxylipins have been revealed in SLE patients by lipidomic analysis (122, 123). Oxylipin is a series of oxidative metabolites produced by polyunsaturated fatty acids (PUFAs), such as arachidonic acid (AA), linoleic acid (LA), and alpha-linolenic acid (ALA), that undergo non-enzymatic or specific enzymatic [lipoxygenase (LOX) and cytochrome P450 (CYP450)] oxidations. Multiple oxylipins, such as 12-hydroxy-heptecotrienoic acid (12-HHTrE) and prostaglandin E1 (PGE1), are critical for SLE pathogenesis and have been identified as biomarkers for SLE pathogenesis (124, 125). Interestingly, the concentration of serum PUFAs is significantly decreased in SLE patients, which demonstrates that the antioxidant system is impaired and that lipid peroxidation is prevalent (107, 124). Moreover, lysophospholipids containing PUFA chains and lipid peroxidation substances (i.e., 4-hydroxyalkenals) strongly accumulate in the peripheral blood mononuclear cells of SLE patients (126). In summary, lipid metabolic disorder is one of the outstanding features in SLE, especially pertaining to oxidized-lipid metabolism.

Moreover, lipid metabolic disorder is essential for ferroptosis, because the executor of this cell death is lipid peroxidation, which triggers ion flux disturbances and ultimately plasma membrane permeabilization and fragmentation (127–129). Lipid metabolism is crucial for the initiation, propagation and termination of lipid peroxidation (130). PUFAs [especially AA (C20:4) and LA (C18:2)] are regarded as primary inducers of ferroptosis because whose bis-allylic hydrogens have relatively low bond dissociation energies and are more sensitive to lipid peroxidation (19, 131). In brief, the contributions of PUFAs to ferroptosis can be divided into two main categories: extension into the membrane and peroxidation. First, PUFAs are taken into the cell or converted by other lipids inside the cell. Then, extension can occur into membrane phospholipids (PUFA-PLs) via acyl-CoA synthetase long-chain family member 4 (ACSL4) and lysophosphatidylcholine acyltransferase 3 (LPCAT3), which are very vulnerable to peroxidation. Second, PUFA-PLs are oxidated by lipoxygenases or via nonenzymatic autoxidation reactions [such as the Fenton reaction). The lipid peroxidation chain reaction is subsequently maintained by lipid radicals (such as the phospholipid peroxyl radical (PLOO·)] derived from lipid peroxidation. When antioxidative agents, including GPX4 and coenzyme Q10 (CoQ10), are depleted, the cell ultimately undergoes ferroptosis (132).

**Figure 3:**
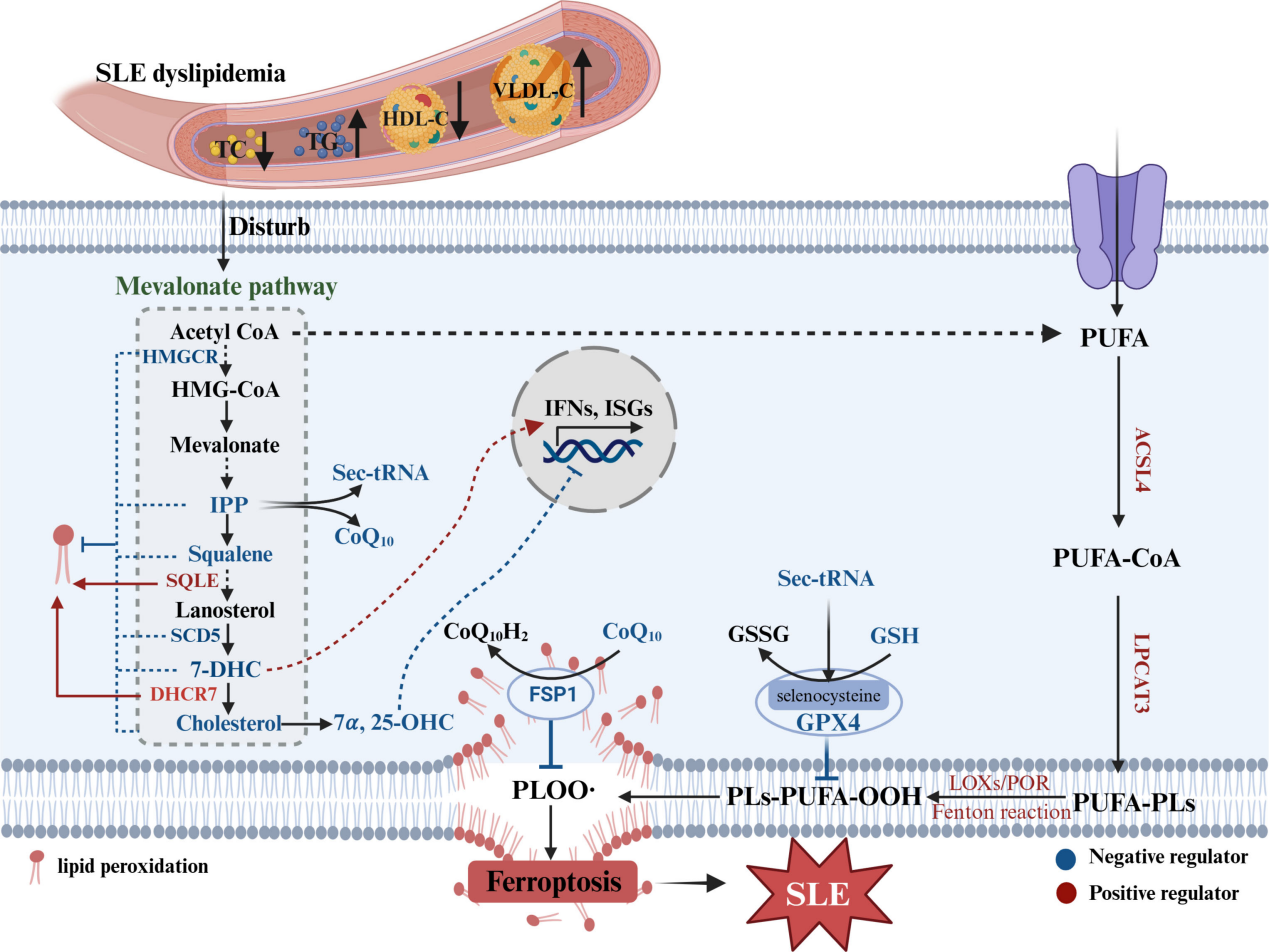
Lipid metabolism disorder in SLE and ferroptosis. SLE dyslipidemia can disturb cholesterol metabolism, which intermediates or enzymes not only affect IFNs response but mediate ferroptosis. Sec-tRNA, selenocysteine tRNA required for GPX4 synthesis; TC, total cholesterol; TG, triglyceride; HDL-C, high density lipoprotein-cholesterol; VLDL-C, very low density lipoprotein-cholesterol; HMGCR, 3-hydroxy-3-methylglutaryl-coenzyme A reductase; IPP, isopentenyl pyrophosphate; SQLE, squalene monooxygenase; CoQ10, coenzyme Q10; 7-DHC, 7-dehydrocholesterol; DHCR7, 7-dehydrocholesterol reductase; SCD5, sterol C5-desaturase; 7α,25-OHC, 7α, 25-dihydroxycholesterol; FSP1, ferroptosis suppressor protein 1; ISGs, interferon-stimulated genes; LOXs, lipoxygenases; POR, cytochrome P450 reductase; PLs, phospholipids; ACSL4, acyl-CoA synthetase long-chain family member 4; LPCAT3, lysophosphatidylcholine acyltransferase 3.

Although the direct relationship between SLE and ferroptosis related to lipid metabolism has not been reported, there are numerous potential connections as follows: i.The dyslipidemia feature of SLE further promotes cholesterol metabolic dysfunction, which can affect ferroptosis. The intermediate, isopentenyl pyrophosphate (IPP), is required for the synthesis of GPX4 (133, 134) and CoQ10 (135–137), which are known to prevent ferroptosis. Similarly, squalene (138, 139) and 7-DHC (140, 141) effectively prevent lipids from autoxidation and subsequent fragmentation (142). Furthermore, some enzymes related to cholesterol metabolism serve as potential suppressors of ferroptosis, such as 3-hydroxy-3-methylglutaryl-coenzyme A reductase (HMGCR) in mitochondria (143) and sterol C5-desaturase (SCD5), whereas 7-dehydrocholesterol reductase (DHCR7) (140) and squalene monooxygenase (SQLE) (138) function as pro-ferroptostic molecules (3). Elevation of the cellular cholesterol content specifically restrains elevated lipid peroxidation and reduces susceptibility to ferroptosis by decreasing membrane fluidity (138). ii.PUFA, an inducer of ferroptosis, is significantly decreased in the extracellular space but increased in the intracellular space in SLE patients (**Figure 3**). Moreover, the levels of lipid peroxidation substances, especially MDA and 4-HNE (ferroptostic biomarkers), are markedly increased in SLE patients. Moreover, elevated MDA was related with multiple lupus manifestations (such as vasculitis, musculoskeletal, cutaneous and nephritis) (144–146). And also, the serum levels of anti-MDA IgG antibody positively were related with disease activity, active nephritis, inflammatory indictors and the consumption of complement factors (147). This phenomenon may be explained by the fact that PUFAs are taken into cells to be involved in ferroptosis in SLE patients.

### 3.4 Function of DAMPs

#### 3.4.1 HMGB1

DAMPs are endogenous molecules that are released into the extracellular space under some pathologic conditions, mainly during cell death. Although numerous DAMPs released from cell death or necrosis, including TNF-α (148, 149), IL-1β, IL-18 (150, 151) and IL-33 (152), have been identified as biomarkers for SLE, their potential role in SLE has not been well defined.

However, extracellular HMGB1, a ubiquitous DAMP, belongs to the HMG family and performs well in SLE pathogenesis. HMGB1 was originally thought to be located only in the nucleus as a DNA chaperone and participated in regulating chromatin structure and function; however, recently, it was found that it can also be located in the cytoplasm and cell membrane. In addition, HMGB1 can be secreted actively or passively into the extracellular space to perform various biological functions (153, 154). In recent years, much more attention has been given to its extracellular function. A growing number of reports have suggested that extracellular (both urine and plasma) HMGB1 can serve as a biomarker for SLE, which could predict disease activity and renal involvement (152–157). Moreover, anti-HMGB1 antibodies in plasma have also been identified as biomarkers of SLE (158, 159). Extracellular HMGB1 can bind to numerous receptors, such as toll-like receptors (TLRs), and advanced glycosylation end-product-specific receptor (AGER), triggering receptor expression on myeloid cells-1 (TREM1), which promotes the release of pro-inflammatory factors. In addition, when released outside the cell, HMGB1 usually binds to intranuclear material (e.g., RNA, DNA), which can activate multiple intracellular nucleic acid sensors, including TLR7, TLR9 and cGAS (160, 161). These sensors are tightly correlated with increased IFN production and SLE pathogenesis (162, 163). In addition, both I-IFN and II-IFN can promote HMGB1 release, resulting in a vicious cycle (24, 164), which may aggravate the progression of SLE. Overall, numerous results suggest that extracellular HMGB1 is involved in the pathogenesis of SLE. Similarly, treatment with an HMGB1 monoclonal neutralizing antibody can alleviate the phenotype of MRL/*lpr* and BXSB Lupus mice, but it has also been shown to be ineffective in MRL/*lpr* mice (20).

Extracellular HMGB1 is derived mainly from cell death, including apoptosis, necroptosis and ferroptosis (165). Various ferroptostic agonists (including erastin and RSL3) can increase HMGB1 release, and this process is blocked by ferroptostic antagonists (such as ferrostatin-1 and liproxstatin-1) or pro-ferroptostic genetic deficiency (e.g., Acsl4 shRNA) in cancer and noncancer cells (21). Wiernicki B et al. recently demonstrated that ferroptosis can be clearly distinguished into three stages: (1) At the beginning, there is intracellular lipid peroxide accumulation; (2) In the intermediate stage, cell membrane permeability increases, resulting in ATP release; and (3) In the final stage, there is complete disintegration of the cell membrane, and HMGB1, LDH and inflammatory factors are released (166). HMGB1 serves as the main DAMP of ferroptosis and mediates the pathological effects of ferroptosis. It is released during ferroptosis of M2 macrophages and can promote the inflammatory response of M1 cells through the TLR4/STAT3 pathway. Moreover, the inhibition of ferroptosis rescues this effect (167). Wang CB et al. revealed that the hepatotoxic effect of methotrexate (MTX) was mainly mediated by HMGB1 released from ferroptostic cells (31). In addition, Zhang DF et al. reported that renal dysfunction related to imidacloprid was primarily attributed to HMGB1 release from ferroptosis, which promoted the activation of the nucleotide-binding domain (NBD), leucine-rich repeat (LRR), and pyrin domain (PYD)-containing protein 3 (NLRP3) inflammasome in neighboring cells through the RAGE/TLR4-NF-κB signaling pathway, further aggravating pyroptosis (22). Interestingly, numerous recent reports have shown that HMGB1 released from ferroptosis can also trigger ferroptosis in neighboring cells, thus forming a vicious cycle and aggravating the progression of the disease. Wei Q et al. revealed that ultraviolet B radiation induces autophagy-dependent ferroptosis and that subsequently released HMGB1 can promote ferroptosis in neighboring cells through TfR1 (32). Davaanyam D et al. demonstrated that ferroptosis occurs in neurons at the early stage of cerebral ischemia and that HMGB1 can upregulate the expression of hepcidin through TLR4/C-X-C chemokine receptor type 4 (CXCR4) to promote ferroptosis and aggravate cerebral ischemia. Moreover, inhibition of HMGB1 can intercept ferroptosis and alleviate cerebral ischemia (23). In addition, Deng YL et al. reported that the overexpression of casepase-6 in THP-1 cells promoted ferroptosis in HTR8/SVneo cells. However, HMGB1-neutralizing antibodies can prevent this process (168). Taken together, these findings indicate that HMGB1 is an important effector of ferroptosis that can mediate the involvement of ferroptosis in various pathological mechanisms and disease progression.

**Figure 4:**
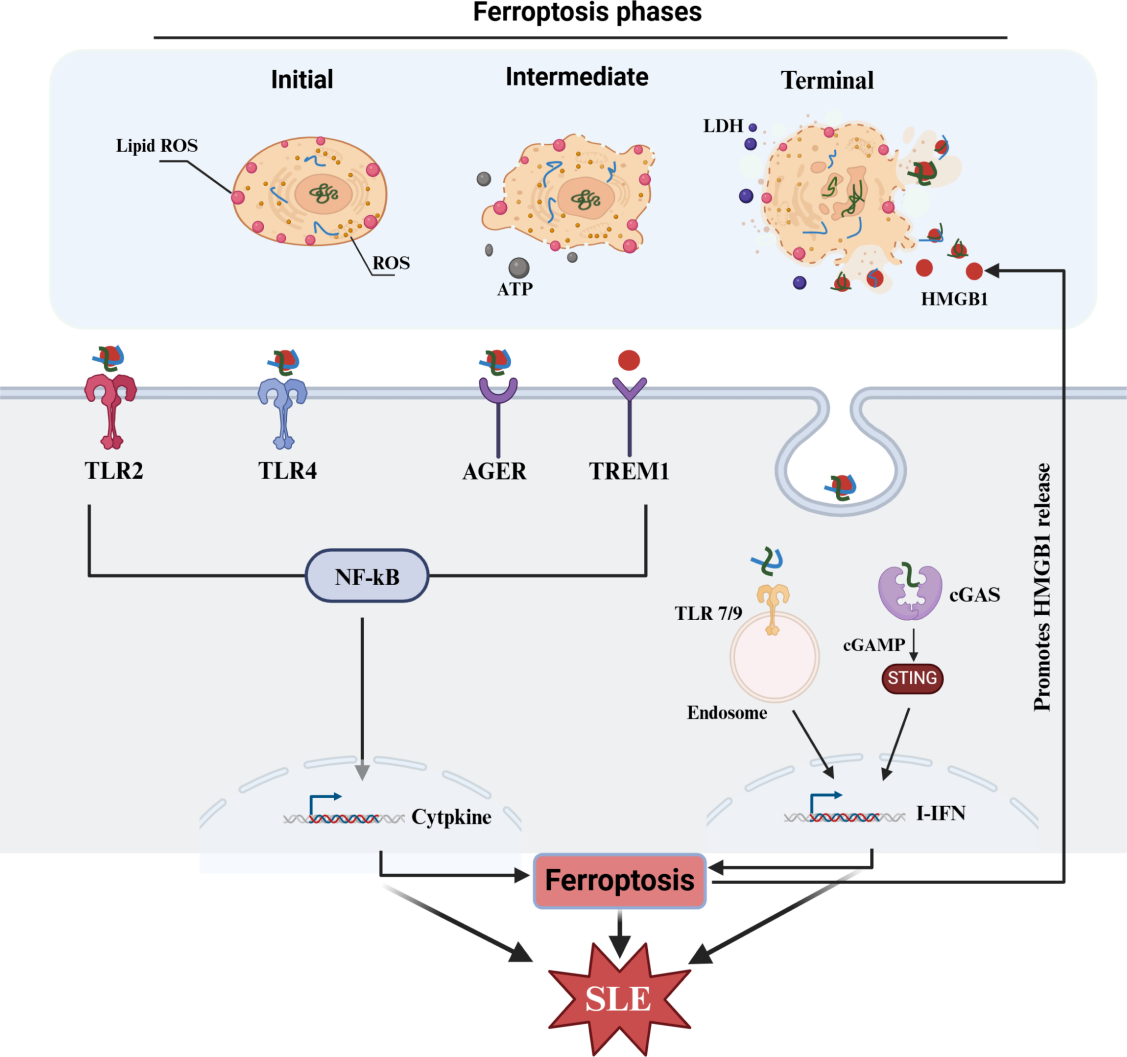
HMGB1 in SLE and ferroptosis. HMGB1 as a major DAMPs released from ferroptosis can intensify the production of IFNs and other cytokines, which subsequently contribute ferroptosis and SLE progress. HMGB1, high-mobility group box 1; TLR, toll-like receptors; AGER, advanced glycosylation end-product-specific receptor; TREM1, triggering receptor expressed on myeloid cells-1; cGAS, cyclic GMP-AMP synthase; STING, stimulator of interferon genes; cGAMP, cyclic GMP-AMP; ISGs, interferon-stimulated genes.

In summary, ferroptosis can increase the release of HMGB1, which can intensify the release of inflammatory factors through its sensors. Additionally, binding to nuclear substances can activate related nucleic acid receptors (TLR7/9, cGAS, etc.) to mediate the mass production of IFNs, and subsequently, IFNs can promote ferroptosis and accelerate the release of HMGB1. Moreover, HMGB1 can further promote ferroptosis in neighboring cells. Overall, these events form a vicious cycle, which may exacerbate the progression of SLE (**Figure 4**).

#### 3.4.2 IFNs

SLE is known as an “interferon signature” disease and the overactivation of the interferon system is closely related to SLE pathogenesis, especially I-IFN, which is elevated before the onset of SLE and closely associated with disease activity and organ involvement (169–171). Nucleic acid substances bind to the endosomal or intracellular nucleic acid receptors (TLRs, cGAS, Nucleotide-binding and leucine-rich repeat receptors (NLRs), etc.) and promote the massive production of interferon-stimulated genes (ISGs) and Type I IFNs (I-IFNs), subsequently contributing to SLE progression. In the past, type I IFNs (II-IFNs) were considered to play the most crucial role in the pathogenesis of SLE (169). Recently, other types of IFNs, especially II IFNs, have also been confirmed to play an important role in the pathogenesis of SLE (170, 171). Moreover, in recent years, IFNs have been shown to be crucial for the regulation of ferroptosis (8). Li PC et al. reported that, in SLE, IFN-α or IgG can restrain GPX4 transcription through CaMKIV/CREMα, which promotes an increase in the level of intracellular lipid ROS and ultimately facilitates neutrophil ferroptosis. Moreover, they confirmed that neutrophil ferroptosis is a key form of cell death involved in the pathogenesis of SLE (172). In addition, evidence that IFN-α can induce ferroptosis was confirmed by Zhang SL et al. (173). They reported that manganese could disturb the expression of DHODH by activating cGAS-STING pathway, promoting an increase in mitochondrial lipid peroxides and ROS, ultimately leading to ferroptosis of tumor cells. Zhang SL et al. also found that blocking IFNAR could rescue this effect of manganese. As the downstream pathway of IFNs, the JAK-STAT pathway is considered one of the most important pathways involved in the pathogenesis of SLE (mainly JAK-STAT1) (174, 175). In addition, many JAK inhibitors (such as tofacitinib, baricitinib, upadacitinib and filgotinib) are being investigated in clinical trials (176). Recent studies have confirmed that spermine can effectively alleviate the progression of SLE by inhibiting JAK1 (177).

Moreover, a close correlation between the IFNs-JAK-STAT pathway and ferroptosis has been demonstrated in recent years. In another autoimmune disease, Sjogren’s syndrome, high expression of IFN-γ could decrease the expression of solute carrier family 3 member 2 (SLC3A2, a subunit of xCT), glutathione, and GPX4 in salivary gland epithelial cells and then trigger ferroptosis through activation of the JAK/STAT1 pathway, which can be inhibited by JAK1/2 or STAT1 antagonists (178). In addition, in retinal pigment epithelial cells, IFN-γ can downregulate the expression of solute carrier family 40 member 1 (SLC40A1, iron export protein) and SLC7A11 through the JAK1-2/STAT1 pathway, which promotes the accumulation of intracellular Fe^2+^ and the exhaustion of glutathione and ultimately induces ferroptosis (179). In tumor cells, IFN-γ can also prevent the transcription of both SLC3A2 and SLC7A11 and then accelerate ferroptosis via JAK1/2-STAT1-IRF1 (180). Furthermore, a chromatin immunoprecipitation assay confirmed that IFN-γ promoted the binding of STAT1 to the SLC7A11 promoter to intercept its transcription (181). However, it has also been reported that inhibition of STAT1 can downregulate GPX4 and SLC7A11 expression to promote ferroptosis (182). Fan Li also confirmed that STAT1 can bind to the promoter region of SLC7A11 and promote its transcription using a dual luciferase reporter assay (183). These results suggest that STAT1 can regulate ferroptosis, but the regulatory mechanism is contradictory and may be related to differences in cells and their environment.

**Figure 5:**
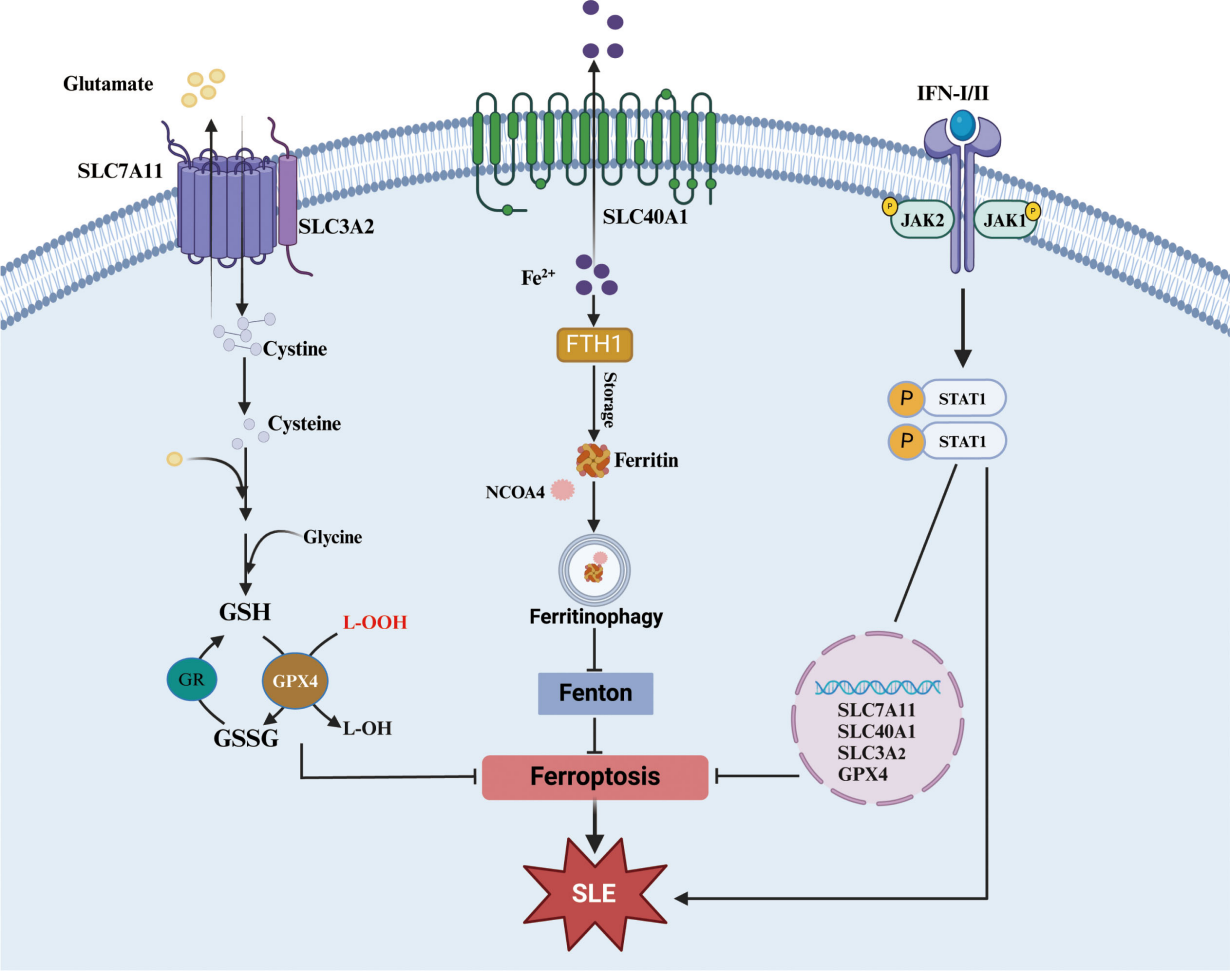
JAK-STAT1 pathway in ferroptosis. Both xCT-GPX4 and FTH1-Ferritinophagy are important roadways to against ferroptosis. And the accumulation of some cytokines (especial I/II-IFN) could significantly affect pivotal molecules of the two roadways although JAK-STAT1 pathway. xCT, cystine/glutamate transport system; SLC7A11, solute carrier family 7a member 11, SLC3A2, solute carrier family 3 member 2; SLC40A1, solute carrier family 40 member 1; GPX4, glutathione peroxidase 4; GSH, glutathione; GSSG, glutathione disulfide; FTH1, ferritin heavy chain 1; NCOA4, nuclear receptor coactivator 4.

In conclusion, JAK-STAT1 not only play important roles in the pathogenesis of SLE but also regulate ferroptosis. Since the regulation of IFNs-JAK-STAT signaling is inconsistent in ferroptosis, whether this signaling is implicated in SLE pathogenesis by regulating ferroptosis needs more evidences (**Figure 5**). Although many inhibitors targeting the JAK-STAT pathway have been investigated in clinical trials, their actual effects are not satisfactory (176). Notably, whether the inhibition of the JAK-STAT pathway potentially aggravates ferroptosis and promotes the progression of SLE deserves further explore.

## 4 Promising therapeutical reagents for SLE by inhibiting ferroptosis

Given that several pathophysiological features of ferroptosis, including iron accumulation, mitochondrial dysfunction, overproduction of lipid peroxidation and suppression xCT-GPX4 pathway are potentially implicated in SLE progression, reversing those features may be beneficial to SLE disease. Below, we summarize some the bioactive pharmacological agents that can against those ferroptostic process (**Table 2**).

**Table 2:** The promising therapeutical reagents for SLE by inhibiting ferroptosis.

Targeted pathway	Reagent	Proposed mechanism	Reference
Iron homeostasis	Deferiprone (DFP)	Iron chelator	(47, 121)
	Dexrazoxane (DXZ)	Iron chelator	(123)
	Deferoxamine (DFO)	Iron chelator	(184, 185)
	Hepcidin	Reducing iron accumulation	(47)
Mitochondria dysfunction	Sirolimus	an mTOR inhibitor and recovering mitochondrial dysfunction	(186–189)
	Metformin	normalizing mitochondrial dysfunction	(190, 191)
	MitoQ	Analogue of CoQ10, special for preventing mtROS	(86, 192)
Lipid peroxidation	Thiazolidinediones (TZDs)	Selectively inhibiting ACSL4	(193, 194)
	Liproxstatin-1 (Lip-1)	Special ferroptostic inhibitor	(172)
	CoQ10	A potent radical-trapping antioxidant and inhibiting lipid peroxidation	(195)
	Idebenone	Analogue of CoQ10	(196)
	Vitamin K	A potent radical-trapping antioxidant and inhibiting lipid peroxidation	(197–199)
xCT-GPX4 pathway	N-acetylcysteine (NAC)	Increasing cysteine levels and facilitating GSH synthesis	(200–202)
	Selenium (Se)	Essential for maintaining GPX4 activity	(79)
	Glycyrrhizin	Increasing GSH and GPX4, decreasing MDA, Fe2+, ROS	(203)

### 4.1 Inhibiting iron accumulation

Intracellular iron accumulation is the chief culprit of ferroptosis and can promote SLE progression, the therapeutical reagents to reduce iron concentration may be beneficial to treat SLE. Iron chelators, including deferiprone (DFP), dexrazoxane (DXZ) and deferoxamine (DFO), are commonly used in clinical. Although only few reports demonstrated DFO could alleviated SLE progression, multiple evidences confirmed those chelators performed an effectivity on some chronical diseases by preventing ferroptosis, such as osteoarthritis (184), I/R-induced injury (185), non-alcoholic steatohepatitis (204), neurodegeneration (205) and so on. Additionally, hepcidin, the iron-regulatory hormone, has been verified it could reduce progression and severity of LN by decreasing renal iron accumulation (47).

### 4.2 Recovering mitochondrial dysfunction

Mitochondrial dysfunction not only drivers ferroptostic process but promotes SLE progression. Recovering mitochondrial dysfunction to prevent ferroptosis may improve prognosis of SLE disease. Recent evidences demonstrated mTOR inhibition suppress ferroptosis by mediating mitochondrial dysfunction (186). Sirolimus is an mTOR inhibitor that server as an antifungal or immunosuppressive agent in clinical, which also effectively alleviates lupus mice manifestation (187, 188). Moreover, Lai ZW et al. demonstrated sirolimus improve SLE patient activity by recovering mitochondrial dysfunction (189). Metformin, commonly used in clinical to treat type II diabetes, can reduces oxidants by normalizing mitochondrial dysfunction (190, 191). Remarkably, it has been verified to improve SLE patients by recovering mitochondrial dysfunction (206–208). Additionally, MitoQ, as an analogue of CoQ10, special for preventing mtROS production, was shown to alleviate lupus mice (86, 192).

### 4.3 Reducing lipid peroxidation

Lipid peroxidation is essential to ferroptosis and prevent the process was shown to improve numerous ferroptosis-related diseases. Thiazolidinediones (TZDs), including rosiglitazone, pioglitazone and troglitazone, are applied to treat type II diabetes. Remarkably, TZDs performed an ability to suppress ferroptosis though selectively inhibiting ACSL4 and reduce mortality in kidney-specific *Gpx4* knockout mice (193, 194). Liproxstain-1 (Lip-1), a spiroquinoxalinamine derivative, was identified as a special ferroptostic inhibitor by high-throughput screening. Recent evidence shown it could alleviate MRL/*lpr* lupus mice symptom (172). Additionally, some lipid antioxidants also display potential therapeutical prospect as follow: i. CoQ10 is a major substrate of FSP1 that known as ferroptostic suppressor. The dietary supplement of it as a potential candidate for the treatment of various noncommunicable diseases (195). Moreover, its analogues, idebenone with better bioavailability and efficacy, was shown to attenuate murine lupus (196). ii. Vitamin K, resembled CoQ10 structure, was used to overcome warfarin poisoning in clinical practice and its supplementation could positively affect multiple chronical diseases (197). Interesting, it also was identified as a lipid radical-trapping antioxidant to prevent ferroptosis via FSP1-mediated pathway (198, 199).

### 4.4 Activating xCT-GPX4 pathway

As described above, the xCT and GSH-GPX4 pathway are identified as primary defense mechanism to ferroptosis. N-acetylcysteine (NAC), a precursor to cysteine, has been shown to prevent ferroptosis by increasing cysteine levels and facilitating the synthesis of γ-glutamyl-cysteine and GSH (200). And its supplement is potentially beneficial for comorbid disorders associated with heavy alcohol consumption (201) and Parkinson’s disease symptoms (202). Micronutrient selenium (Se) is essential for maintaining GPX4 activity. Optimal Se supplement could improve hemorrhagic and ischemic stroke prognosis via inhibiting ferroptosis in mice (79). Additionally, glycyrrhizin, extracted from the glycyrrhiza and commonly used in clinical, could alleviate acute liver failure by increasing GSH and GPX4, decreasing MDA, Fe^2+^, ROS to inhibit ferroptosis (203).

## 5 Conclusion

In summary, SLE is a complex chronic disease for which its pathophysiology and effective treatments remain speculative. Ferroptosis is a prevalent type of pathogenic cell death in chronic disease. Although the studies on ferroptosis in SLE pathogenesis are still in early stages, there are intricate and close connections among them, which provides opportunities for understanding SLE pathophysiology and development of novel therapeutics.
